# Folliculitis might be associated with pathergy-positivity in patients with Behçet syndrome

**DOI:** 10.1097/MD.0000000000037553

**Published:** 2024-03-22

**Authors:** Zeynep Altan Ferhatoğlu, Dursun Dorukhan Altinişik, Ayşe Özdede, Defne Özkoca, Sabriye Güner, Kadir Atacan Yildiz, Zekayi Kutlubay, Emire Seyahi, Vedat Hamuryudan

**Affiliations:** a Department of Dermatology Istanbul, Istanbul University-Cerrahpasa, Cerrahpasa Medical Faculty, Istanbul, Turkey; b Department of Dermatology, Orhangazi State Hospital, Bursa, Turkey; c Department of Internal Medicine, Division of Rheumatology, Istanbul University-Cerrahpasa, Cerrahpasa Medical Faculty, Istanbul, Turkey; d Department of Dermatology, Koç University, School of Medicine, Istanbul, Turkey.

**Keywords:** Behçet syndrome, folliculitis, leg ulcer, pathergy test

## Abstract

Pathergy test indicates nonspecific hyper-reactivity of the skin to aseptic trauma in Behçet syndrome (BS) and is considered as an adjunctive diagnostic test with a good specificity albeit with low sensitivity. We tested the hypothesis that a relationship exists between active clinical manifestations of BS and the pathergy-positivity when performed simultaneously. Pathergy test and detailed dermatologic examination were done in 105 BS patients (60M/45F); who were seen consecutively at the multi-disciplinary BS outpatient clinic in a single tertiary center. Information regarding demographic and clinical characteristics, pathergy test results at diagnosis, and details about treatment were obtained from patient charts. Disease activity was assessed using Behçet Disease Current Activity Form. Among 105 patients, 27 (25.7%) were pathergy-positive at the time of the study visit whereas 40.9% were pathergy-positive at the time of the diagnosis. There was no relation between pathergy test and patient age or disease duration, either. Pathergy-positivity was significantly more common in patients with folliculitis compared to those without folliculitis (40.7% vs 19.2%; *P* = .026). The test was also positive in all 3 patients with leg ulcers due to venous stasis. We found that among all skin-mucosa lesions only the presence of folliculitis was associated with pathergy positivity with statistical significance. It was also remarkable that the current pathergy was positive in all 3 patients with active leg ulcers but this finding warrants further studies because of the low patient numbers.

## 1. Introduction

Behçet syndrome (BS) is a multi-systemic inflammatory disease characterized by recurrent mucocutaneous lesions, arthritis and a sight-threatening uveitis. It can also involve blood vessels of all types and sizes, gastro-intestinal system and central nervous system leading to increased mortality. A peculiar characteristic of BS is the hypersensitivity of the skin to aseptic trauma, named as the pathergy reaction.^[1–4]^ The pathergy reaction can be induced by intradermal pricking the forearm skin usually with 20 gauge sterile needles. The result is accepted as positive if an erythematous based pustule or an indurated erythematous papule is observed at the prick site 24 to 48 hours later. Although its specificity is high, the sensitivity is variable and is influenced by several factors such as ethnicity, gender and disease severity.^[4]^ Furthermore, the pathergy reaction has a low reproducibility and is not constant throughout the disease course.^[5]^ Some previous studies have found a relationship between pathergy-positivity and male gender, oral aphtha, folliculitis and uveitis but a well-established association between the pathergy-positivity and specific manifestations of BS has not been documented.^[6]^ It is also important to note that recent studies have revealed a decreased incidence of pathergy positivity in BS.^[7]^ The aim of this study is to assess the association of actual pathergy positivity when performed simultaneously during the presence of active skin lesions. Our hypothesis was based on the assumption that especially folliculitis would result in increased positivity of the pathergy reaction.

## 2. Patients and method

Consecutive patients attending the BS Research Center at Istanbul University-Cerrahpaşa, Cerrahpaşa Medical Faculty and fulfilling the International Criteria^[2]^ were included in this study between September 2022 and February 2023. The exclusion criteria were pregnancy, lactation, and age younger than 18 years. The approval of Istanbul University-Cerrahpaşa, Cerrahpaşa Medical Faculty Ethics Committee was taken before the initiation of the study (13.09.2022-474654). Patient consent was obtained at the clinical visit.

The age, gender, disease duration, types of organ involvement, human leukocyte antigen B51 (HLA-B51) status, and medication history (conventional or biological immunomodulatory drugs) were retrieved from the patient charts. At the study visit, a dermatologist (Zeynep Altan Ferhatoglu ) performed the dermatological examination after taking the patient history. Behçet Disease Current Activity Form (BDCAF) score (ranging from 0 to 6) was calculated and information about smoking history was obtained. The dermatological examination included the active lesions of oral aphthae (minor, major or herpetiform), genital ulceration, extra-genital ulceration, folliculitis, erythema nodosum-like lesion, Sweet syndrome-like eruptions, superficial venous thrombosis and venous stasis related skin diseases (leg ulcers, stasis dermatitis and post-inflammatory hyperpigmentation). Acneiform lesions were excluded, only eruptions compatible with folliculitis were evaluated. Major oral ulcers were defined as larger than 1 cm in diameter and/or which healed with scarring. Minor oral ulcers were defined as smaller than 1 cm in diameter healing without scar formation.^[8]^ Pathergy test was performed by using a sterile 20 gauge blind-ended needle at 6 points on the forearm by a dermatologist. The pathergy test was evaluated by another dermatologist (Dursun Dorukhan Altinisik.) who was unaware of the study hypothesis. The evolution of a papule or pustule at the test site after 48 hours was accepted as positive.

### 2.1. Statistical analysis

Chi square or Fisher exact test was used to evaluate categorical data. Student *t* test was used to compare continuous variables between study groups. Mann–Whitney *U* test was used for comparison of non-normally distributed variables. Univariate and bivariate logistic regression analyzes were performed to determine the independent risk factors for current pathergy positivity. A *P* value of <.05 was accepted as significant. IBM SPSS Statistics for Windows, v.25.0 (IBM Corp., Armonk, NY, USA) was used in the statistical analysis.

## 3. Results

We interviewed 800 consecutive patients diagnosed with BS who were admitted to the Istanbul University-Cerrahpaşa BS Research Center between September 2022 and February 2023. Pathergy test was performed in 298 patients who agreed to participate in the study. We conducted our study with 105 patients (35.2%) (60M/45F) who came back to the clinic 48 hours later for the evaluation of the pathergy test.

### 3.1. Demographic and clinical characteristics

Demographic and clinical manifestations observed in our patient population are summarized in Table 1. The median age of the patients was 43 (18–76) years and the median disease duration was 10 years (minimum–maximum: 1–47). A total of 40 patients (38.0%) were active smokers. We were able to reach the previous pathergy results at the time of diagnosis in 94 patients from the patient charts and 38 (40.4%) were pathergy positive. Data related with HLA-B51 was present only in 38 patients in whom carrier rate was 60.5%. While, 102 (97.1%) patients had skin-mucosa involvement, significant portion of patients had ocular (45.7%), joint (29.5%) and vascular (24.7%) involvement. Active muco-cutaneous manifestations observed at the time of study were minor aphthae (49.5%), major aphthae (4.5%), genital ulceration (6.7%), folliculitis (24.8%), sweet-syndrome-like eruption (1%), erythema nodosum-like lesion (5.7%), venous stasis related skin disorders (6.7%) and superficial venous thrombosis (1%). Active herpetiform aphthae and extragenital ulcers were not detected in any of the patients. The median BDCAF score was 1 (minimum: 0–maximum: 6). A total of 26 patients (18M/8F) (22.0%) were in inactive stage. At the time of the study, patients were using one or multiple drugs such as colchicine (n = 72), non-biological disease modifying anti-rheumatic drug (DMARD) (n = 40), glucocorticoids (GC) (n = 22), anti-TNF agents (n = 22), and interferon alpha (n = 4). Among 105 patients, 56 (53.3%) were using DMARDs (biological or non-biological) with or without GC. The remaining 49 patients were either using solo colchicine (n = 46) or were off treatment (n = 3).

**Table 1:** Demographic and clinical characteristics of the patients.

	Total n = 105	Males (1) N = 60	Females N = 45	*P*
Age, yr (median) (min–max)	43 (18–76)	42 (20–76)	44 (18–70)	.744*
Disease duration, yr (median) (min–max)	12 (1–47)	13 (1–48)	11 (1–32)	.287*
Smoking, n (%)†				
No	51 (49.5)	19 (32.2)	32 (72.7)	<.001
Yes	40 (38.8)	32 (54.2)	8 (18.2)	
Ex-smoker	12 (11.7)	8 (13.6)	4 (9.1)	
Positive pathergy test at the time of diagnosis, n (%)‡	38 (40.4)	25 (44.6)	13 (34.2)	.312
Positive HLA-B51, n (%)§	23 (62.2)	11 (73.3)	12 (54.5)	.247
Involved System				
Ocular, n (%)	48 (45.7)	29 (48.3)	19 (42.2)	.534
Articular, n (%)	31 (29.5)	23 (38.3)	8 (17.8)	.022
Vascular, n (%)	26 (24.7)	25 (41.7)	1 (2.2)	<.001
Neurological, n (%)	2 (1.9)	2 (3.3)	0	.324
Intestinal, n (%)	2 (1.9)	1 (1.7)	1 (2.2)	.670
Active skin-mucosa lesions				
Minor aphthae, n (%)	52 (49.5)	27 (45.0)	25 (55.6)	.284
Major aphthae, n (%)	5 (4.8)	2 (3.3)	3 (6.7)	.365
Genital ulcer, n (%)	7 (6.7)	4 (6.7)	3 (6.7)	.658
Folliculitis, n (%)	26 (24.8)	18 (30.0)	8 (17.8)	.113
Sweet-syndrome like eruption, n (%)	1 (0.9)	0	1 (2.2)	N/A
Erythema nodosum like lesion, n (%)	6 (5.7)	2 (3.3)	4 (8.9)	.214
Superficial venous thrombosis, n (%)	1 (0.9)	1 (1.7)	0	N/A
Venous stasis related skin disorders (Leg ulcer, SD, PIH), n (%)	7 (6.7)	5 (8.3)	2 (4.4)	.354
Behçet’s disease current activity form (median) (min–max)	2 (0–6)	2 (0–5)	2 (0–6)	.389**
Current treatment with any DMARD with or without GC, n (%)	56 (53.3)	36 (60.0)	20 (44.4)	.114

DMARD = disease modifying anti-rheumatic drugs either biological or non-biological, GC = glucocorticoids, HLA-B51 = human leukocyte antigen B51, PIH = post-inflammatory hyperpigmentation, SD = stasis dermatitis.

† Information about smoking was available in 103 patients.

‡ Information about pathergy results at diagnosis was present in 94 patients.

§ Information about HLA-B51 was available in 38 patients.

* Student’s *t* test.

** Mann–Whitney *U* test.

Male patients had significantly more joint (38.3% vs 17.8%, *P* = .022) and vascular involvement (41.7% vs 2.2%, *P* < .001) compared to females. Additionally, they were more likely to smoke. Besides that, there was no difference between male and female patients regarding age, disease duration, pathergy positivity at diagnosis, HLA-B51 carrier rate, active skin-mucosa lesions, BDCAF score and immunosuppressive use.

### 3.2. Current and previous pathergy tests

Twenty-seven (25.7%) patients had positive pathergy at the time of the study. The rate of current pathergy positivity was higher in men (31.7%) than women (17.8%), however the difference was not statistically significant (*P* = .107). Pathergy test was positive at the time of diagnosis in 38 patients (40.4%). Among these 38 patients, 25 (65.8%) became pathergy-negative at the time of the study. Of the 56 patients with negative pathergy at the time of diagnosis 47 (83.9%) were again negative at the time of the study. Pathergy positivity at the time of the study was significantly higher in patients who had positive pathergy test at the time of diagnosis. (*P* = .042).

### 3.3. Demographic and clinical variables associated with current pathergy positivity

Table 2 shows distribution of demographic and clinical characteristics between current pathergy positive and negative groups. Current pathergy positivity was found to be independent of the patient age and disease duration. No significant relationship was found between pathergy positivity and HLA-B51 status, BDCAF score, major organ involvement or immunosuppressive treatment, either. While there was no association with large vessel involvement, it was found to be positive in all 3 patients with leg ulcers developed due to the venous stasis.

**Table 2:** Comparison of demographic and clinical characteristics between current pathergy positive and negative patients

	Current pathergy positive N = 27 (19M/8F)	Current pathergy negative N=78 (41M/37F)	*P*
Age, years (median) (min–max)	41 (18–62)	44 (19–76)	.189^**§**^
Gender (being male)	19 (70.3)	41 (52.6)	.107
Disease duration in years (median) (min–max)	10 (1–24)	10 (1–47)	.834^**‖**^
Smoking, n (%)*			
No	14 (51.9)	37 (48.7)	.726
Yes	11 (40.7)	29 (38.2)	
Ex-smoker	2 (7.4)	10 (13.2)	
Positive pathergy test at the time of diagnosis, n (%)†	13 (48.1)	25 (32.0)	**.042**
Positive HLA-B51, n (%)‡	6 (22.2)	17 (21.7)	.749
Involved organ system			
Ocular, n (%)	11(40.7)	37 (47.4)	.547
Articular, n (%)	7 (25.9)	24 (30.8)	.634
Vascular, n (%)	8 (29.6)	18 (23.1)	.497
Neurological, n (%)	0	2 (2.6)	N/A
Intestinal, n (%)	0	2 (2.6)	N/A
Behçet’s disease current activity form (median) (min–max)	1(0–4)	1(0–6)	.798^‖^
Treatment with any DMARD (biologiocal or non–biological) with or without GC, n (%)	17 (62.9)	39 (50.0)	.245

DMARD = disease modifying anti-rheumatic drugs either biological or non-biological, GC = glucocorticoids.

* Information about smoking was available in 103 patients.

† Information about pathergy results at diagnosis was present in 94 patients.

‡ Information about HLA-B51 was available in 38 patients.

§ Student’s *t* test.

‖ Mann–Whitney *U* test.

Examining the relationship between active skin-mucosa lesions and current pathergy status, we found that only the presence of active folliculitis was significantly associated with current pathergy positivity (Table 3). Among 24.7% of the patients with active folliculitis the current pathergy positivity was 40.7% (*P* = .027; *χ*^2^ test). In univariate logistic regression analysis presence of folliculitis was a predictable factor for pathergy positivity (*P* = .029; OR: 2.88 [95% confidence interval 1.1–7.5]). Both the positivity of current pathergy and active folliculitis were more common in men than in women with no statistical difference (men vs women, 31.7% vs 17.8%, *P* = .107 for pathergy and 30% vs 17.8%, *P* = .151 for folliculitis). In bivariate analysis, folliculitis and pathergy positivity were gender independent (Table 4).

**Table 3:** Comparison of the active skin-mucosa lesions and the current pathergy status.

Active skin-mucosa lesions	Current pathergy positive N = 27 (19M/8F)	Current pathergy negative, N = 78 (41M/37F)	*P* value
Minor aphthae, n (%)	15 (55.6)	37 (47.4)	.467
Major aphthae, n (%)	2 (7.4)	3 (3.8)	.454
Genital ulcer, n (%)	2 (7.4)	5 (6.4)	.858
Folliculitis, n(%)	11 (40.7)	15 (19.2)	.027
Sweet-syndrome like eruption, n (%)	0	1 (1.3)	N/A
Erythema nodosum like lesion, n (%)	2 (7.4)	4 (5.1)	.660
Superficial venous thrombosis, n (%)	1 (3.7)	0	N/A
Venous stasis related skin disorders (Leg ulcer, SD, PIH), n (%)	4 (14.8)	3 (3.8)	.07

PIH = post-inflammatory hyperpigmentation, SD = stasis dermatitis.

**Table 4:** Bivariate logistic regression analyses for current pathergy positivity.

Risk Factors	*P*	Odds ratio	95% CI for EXP(B)	
			Lower	Upper
Presence of Folliculitis	.047	2.66	1.01	6.98
Gender	.180	0.51	0.19	1.35

CI = confidence interval.

## 4. Discussion

Our cross-sectional study revealed that folliculitis was an independent risk factor for pathergy-positivity, when patients were simultaneously examined. Pathergy test was not found to be associated with the patient age, disease duration, systemic organ manifestations, disease activity or HLA-B51 status. Our study also indicated that pathergy response was not constant throughout the disease course. Furthermore, male patients were more likely to have positive pathergy results, albeit not significantly.

The association between pathergy test and clinical manifestations has been previously investigated in a number of studies yielding variable results.^[3,4,6,7]^ Our results are in line with several studies that reported a strong association with folliculitis/papulopustular lesions^[6,9,10]^ and no association with patient age, disease duration, disease activity or systemic organ involvement.^[11,12]^ On the other hand, different from what we have found, significant relationships between uveitis,^[6]^ vascular involvement^[13]^ and central nervous system involvement particularly among females^[9]^ were observed. An association with oral aphthae, pseudofolliculitis and uveitis especially in males has been also noted.^[6]^ In our study however, the only active mucocutaneous manifestation which had a relationship with pathergy was folliculitis.

Furthermore, the current pathergy test results were positive in all 3 patients with a leg ulcer occurred as a complication of long-standing venous thrombosis. Both macroscopic appearance and the pathophysiology of leg ulcers in Behçet patients resemble skin ulcers seen in pyoderma gangrenosum,^[14]^ one of the rare types of neutrophilic dermatoses along with Sweet syndrome in which pathergy phenomenon may be observed. Therefore, the strong response to pathergy among those with venous ulcers deserves further investigation.

Alli et al reported that both pathergy positivity and folliculitis were more common in male patients^.[13]^ This may raise the question of a confounding effect of gender on the relationship between pathergy-positivity and folliculitis. Although a consistent relationship could not be established between BS severity and pathergy positivity, it has been reported that severe disease course and pathergy positivity are more common in males.^[15–17]^ This was also confirmed by genetic risk score analyzes.^[18]^ Similarly, in our study, albeit not statistically significant, male patients were more likely to have positive pathergy results.

We found that the current pathergy test became negative in 25 (65.7%) of 38 patients who were pathergy positive at the time of diagnosis, whereas it became positive in 9 of 56 patients (16.0%) who had a negative pathergy at the time of diagnosis. In parallel with Ergun article, our findings supported that the pathergy test is not constant throughout the disease course.^[4]^ The change in test results was mostly in the direction of from positivity to negativity. This can be explained by the decrease of disease activity over time or due to the effect of immunosuppressive treatment. Yazici et al had observed no correlation between the pathergy reaction and clinical severity.^[15]^ Similarly, in the current study, the reactivity to pathergy test appeared to be irrelevant of disease activity. However, several authors repeated the test in both active and inactive stages of the disease and observed significantly higher positivity rates in patients with active stages.^[19–21]^ In this regard, considering 22.0% of our cohort being in inactive stage and 97.1% being under treatment with either solo colchicine (43.8%) or immunosuppressive agents (53.3%) at the time of current pathergy testing, the decrease in positive response we are observing would be most likely explained by the decrease in disease activity. The amelioration of today conditions compared to the past could be another reason, as well.

The rate of pathergy-positivity in BS varies among countries with higher positivity rates in endemic regions.^[4]^ The positivity in Turkish population, for instance may reach as high as 98%.^[5,9]^ Apart from genetic and environmental circumstances, the heterogeneity of disease phenotypes in different populations has been also suggested as a possible explanation for this variation.^[4]^ Along with the variability of pathergy test among different ethnic populations, the test result may be influenced by the socio-economic status of the patient as well as the recent improvement in sanitation, introduction of disposable needles and increased quality of life, all leading to a decline in the sensitivity of pathergy positivity over time.^[7,22,23]^

Our study has several limitations. We were able to complete our study with a small sample size that may have caused type II statistical error. We think that this is related to the fact that the main component of our study, the pathergy test, is a minimally invasive method and has difficulties such as returning to the hospital 48 hours later. Because of it, only patients who consented and living in the close proximity of our center could be studied. Although we interviewed approximately 800 patients at the beginning, 298 gave consent for the skin prick test. Ultimately, only 105 (13.1%) who came back for reevaluation were studied. Furthermore, patients were not standardized regarding disease duration or medical treatment. A total of 53.3% of the patients were using DMARD or GC GC, while 43.8% were using colchicine alone. These drugs may have affected pathergy responsiveness. The duration of cumulative drug use and the effects of multi-drug using could not be evaluated. Finally, we could not assess the effect of infrequent manifestations of BS on pathergy test because of low numbers.

On the other hand, our study also has strengths. Unlike most studies in the literature that evaluated the pathergy status at the time of diagnosis, we examined simultaneously the relationship between current pathergy status and the active skin-mucosa lesions of the patients. Dermatological examination and pathergy test evaluation were done by 2 independent dermatologists. The latter was blind to the hypothesis of the study. The fact that acneiform eruptions are excluded and only folliculitis were considered is another strength of our study. Finally, since the information related with the pathergy test at diagnosis was available in the majority (94/104), we were able to see the transition in the pathergy responsiveness over time.

## 5. Conclusions

This study supports the previous literature in that pathergy-positivity in BS is independent of the patient age, disease duration and disease activity. In alignment with the previous literature, this study also supports that folliculitis is an independent risk factor for pathergy-positivity. Our results may have diagnostic implications. The skin-prick test has a great diagnostic value however is an invasive test with low reproducibility and decreasing sensitivity among many other technical difficulties. We suggest that a proper and precise dermatological examination revealing active folliculitis could be a match for the positive pathergy test. Further studies should investigate whether dermatological detection of folliculitis may replace or account for it.

## Author contributions

**Conceptualization:** Zeynep Altan Ferhatoğlu, Zekayi Kutlubay, Emire Seyahi, Vedat Hamuryudan.

**Data curation:** Zeynep Altan Ferhatoğlu, Dursun Dorukhan Altinişik, Ayşe Ozdede, Sabriye Guner, Kadir Atacan Yildiz.

**Formal analysis:** Zeynep Altan Ferhatoğlu, Emire Seyahi, Vedat Hamuryudan.

**Investigation:** Zeynep Altan Ferhatoğlu, Dursun Dorukhan Altinişik, Ayşe Ozdede, Sabriye Guner, Kadir Atacan Yildiz.

**Methodology:** Zeynep Altan Ferhatoğlu, Kadir Atacan Yildiz, Emire Seyahi, Vedat Hamuryudan.

**Resources:** Zeynep Altan Ferhatoğlu.

**Supervision:** Zekayi Kutlubay, Emire Seyahi, Vedat Hamuryudan.

**Validation:** Zeynep Altan Ferhatoğlu, Zekayi Kutlubay, Emire Seyahi, Vedat Hamuryudan.

**Visualization:** Zeynep Altan Ferhatoğlu, Defne Özkoca.

**Writing – original draft:** Zeynep Altan Ferhatoğlu, Defne Ozkoca.

**Writing – review & editing:** Zeynep Altan Ferhatoğlu, Zekayi Kutlubay, Emire Seyahi, Vedat Hamuryudan.
